# Post-hysterectomy vaginal haemorrhage: a case report

**DOI:** 10.4076/1757-1626-2-7195

**Published:** 2009-09-17

**Authors:** Zacharoula Sidiropoulou, António Setúbal, Catia Acosta, Eduardo Roberto

**Affiliations:** 1 Department of Gynaecology, Hospital Da Luz, 1500, Lisbon, Portugal

## Abstract

Vaginal endometriosis is characterized by the presence of endometrial tissue in the vagina. In this paper the authors present an unusual case of post-hysterectomy vaginal cuff endometriosis.

## Introduction

Vaginal endometriosis is characterized by the presence of endometrial tissue (endometrial epithelium, glands and stroma) in the vagina. It constitutes an uncommon site of endometriotic implants to occur. We report an unusual case of vaginal cuff endometriosis occurred in a woman after being submitted to TLH for multiple uterine myomas.

## Case presentation

A 42 years old Caucasian woman of Portuguese nationality, G0 P0, with irregular menstruation cycles has been submitted to evaluation for abnormal menstrual bleeding and hypogastric complaints in the past 3 years.

Ultrasonography reported uterus of 12.4 × 10.6 × 6.1 cm, with multiple nodules, the bigger one of 13.0 cm diameter.

The patient underwent surgery; a total laparoscopic hysterectomy (TLH) with morcelation of the uterus before the vaginal extraction, has been effectuated in 115 min time lap with a 3 cm left lateral laceration of the vaginal wall as complication, sutured at the end of the surgery. This complication was due to the fact that the vagina was very narrow and the need for manipulation very hard. The into-hospital stay was 48 hours with no post-operative complications.

The anatomopathology report identified a white, hard, fasciculate tumour of 13.0 cm of major diameter without necrotic or haemorrhagic areas, well delimitated from the resting myometrium and that has been classified as a leiomyoma. The rest of the uterus presented atrophic endometrium and no other alterations have been observed. Observation of the patient two months later of the surgery, reported a well fixed vaginal cuff without granulations, nodularity or residual scars.

**Figure 1:**
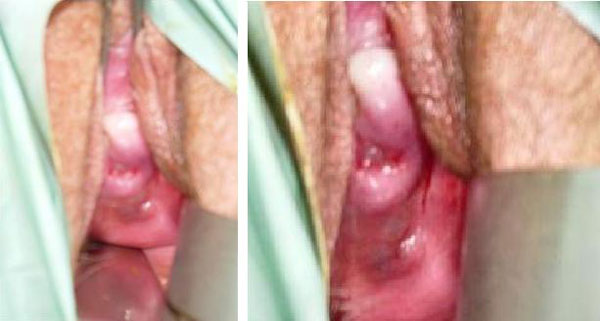
**Yellowish lesions on the vaginal cuff**.

13 months later, the patient referred to her family physician menstrual bleeding. Gynaecological observation revealed yellowish spots of approximately 3 mm on the vaginal cuff, at the area of the TLH suture of the cuff, compatible macroscopically with endometriotic nodular lesions (Figure 1).

The patient underwent surgery with complete excision of the lesion and the histological report referred an endometriotic lesion of the vaginal cuff.

## Discussion

Endometriosis is a well defined but not such well understood benign gynaecological condition that affects 10-15% of reproductive age woman (Kovacs P. Conference Report 2002) and 20-30% of woman undergoing laparoscopy for infertility or chronic pelvic pain, while it has an estimated prevalence of 2-22% in asymptomatic woman [1].

Vagina constitutes a rare localization of endometriotic lesions, usually from spreading of rectovaginal septum of Douglas-pouch initially located lesions (Type I profound endometriosis); sometimes it derives from the dissemination of endometrial tissue during surgical operations like colpoperineoplasty or perineorrhaphy; in other cases (even more rare) it is verified after a laceration during delivery of the vagina, when endometrium has been transformed in decidua.

Excluding the surgical dissemination, in the other cases the endometriotic lesion is located in the 1/3 medium or low of the vaginal wall. In the Douglas-pouch dissemination cases it is localized on the posterior vaginal fornix and it assumes nodular, superficial or profound form, usually identified by digital exam.

The major symptom of vaginal endometriosis is constituted by deep dyspareunia, accentuated in the perimenstrual face.

The current theories about the pathogenetic mechanism of endometriosis include:

1.Â Metastatic theory, with tubaric retrograde flow of menstrual endometrial tissue into the peritoneal cavity, advanced by Sampson in 1927. That seems to be the most widely accepted nowadays.

2.Â Lymphatic and vascular spread of endometrial tissue, where for example, ovarian endometriosis is due to lymphatic spread (Ueki 91).

3.Â Coelomatic metaplasia, with transformation of coelomatic epithelium, present in various organs and tissues into endometrial tissue under the influence of estrogens (Metzger 1991).

4.Â Immunitary system alterations where humoral antibodies to endometrial tissue, aromatase enzymatic expression and various adhesion molecules appear to play an important role on the adherence of ectopic endometrial tissue implants (Zeitoun 1999).

5.Â Surgical dissemination.

The case we report is peculiar in the sense that our patient presented no complaints that constitute the typical symptoms of endometriosis as reported in the literature (cyclic pelvic pain, dysmenorrhoea, menorrhagia, dyspareunia and chronic pelvic pain). She just referred irregularity of the menses, menorrhagia and hypogastric complaints, symptoms that enter on the suspicion of uterine myomas made by ultrasonography and confirmed by the pathology exam that followed the TLH. The preliminary gynaecological observation revealed a straight vagina and a lateral left-side fixation of a globular uterus.

Laparoscopy is considered the major diagnostic approach for endometriosis [2], even though the diagnosis can be only histological on tissue specimens. In this patient, laparoscopy has revealed no signs of endometriosis (macroscopic lesions, circular defects in the peritoneum, endometriomas, and "chocolate" fluid, adnexal or peritoneal adhesions, alteration of pelvic anatomy) even if the complete and extended evaluation of the abdominal cavity has been effectuated as a standard procedure in all our laparoscopic approaches, independently of the reason of the intervention.

13 months after the exeresis, free of any complaints, our patient referred the presence of cyclic menstrual bleeding and the gynaecological observation of endometriosis-like superficial foci has been confirmed by the histology exam on the tissue excised, but we had not the chance to know about the presence of an eventual dyspareunia because of our patient's choice for total sexual abstinence.

The foci we observed were located on the vaginal cuff scar due to the TLH operation, with the typical yellowish nodular aspect, outside the "menstruation" period. There are studies supporting the incidence of endometriosis in post-hysterectomy women which have been negative in laparoscopy and in history [3]. In electronic searching of databases, and abstracts we found similar to our case-reports.

Schram JD [4], reports on 1978, the occurrence of vagina's apex endometriosis 5 years after an abdominal hysterectomy and 4 years after bilateral oophorectomy with no evidence of endometriosis on surgery. Kuhlmann M [5], on 1995 reports two cases, and Grys E [6], on 2001, one case with two years lap time among hysterectomy and endometriosis focus on the vaginal cuff scar. We also have found an article posted on 2003 (Medscape, Management of vaginal endometriosis, 8(1), 2003) of a similar case under the form of a question/communication.

In our opinion, this is a case of surgical dissemination of endometrial tissue which occurred during the extraction of the fragmented uterus via the vagina canal. Yet we cannot exclude the coelomatic metaplasia theory because of the consistent lap-time among the operation itself and the syndromic manifestation.

Both theories have been supported to explain the occurrence of endometriosis after total hysterectomy with conservation of the adnexa.

Finally we cannot also exclude the spread of a microscopic, silent, not primarily observed in laparoscopy, endometriotic focus that forward gave a new localization on the surgical scar.

## Abbreviations

G: gestation; P: parity; TLH: total laparoscopic hysterectomy.

## Consent

Written informed consent was obtained from the patient for publication of this case and accompanying images. A copy of the written consent is available for review by the Editor-in-Chief of this journal.

## Competing interests

The author declares that they have no competing interests.

## Authors' contributions

AS and ER are two major, laparoscopic gynaecological and general surgeons. ZS and CA are surgery residents. The four of them constitute a surgical team dedicated in laparoscopic surgery.
